# Chronotherapy in Oncology: Aligning Cancer Treatment with Biological Time

**DOI:** 10.3390/jpm16080428

**Published:** 2026-08-14

**Authors:** Andrej Belančić, Marin Golčić, Almir Fajkić, Vlatka Bračić, Marko Skelin, Antonio Markotić, Ivan Ćavar, Dragan Trivanović, Ivana Mikolašević

**Affiliations:** 1 Department of Basic and Clinical Pharmacology and Toxicology, Faculty of Medicine, University of Rijeka, 51000 Rijeka, Croatia; markoskelin@yahoo.com; 2 Tumor Clinic, Clinical Hospital Centre Rijeka, 51000 Rijeka, Croatia; marin.golcic@gmail.com (M.G.); ivana.mikolasevic@gmail.com (I.M.); 3 Faculty of Medicine, University of Rijeka, 51000 Rijeka, Croatia; dragan.trivanovic@obpula.hr; 4 Department of Pathophysiology, Faculty of Medicine, University of Sarajevo, 71000 Sarajevo, Bosnia and Herzegovina; almir.fajkic@mf.unsa.ba; 5 Pharmacy Department, General Hospital Šibenik, 22000 Šibenik, Croatia; vlbracic@gmail.com; 6 Department of Physiology, School of Medicine, University of Mostar, 88000 Mostar, Bosnia and Herzegovina; antonio.markotic@mef.sum.ba; 7 Department of Immunology, School of Medicine, University of Mostar, 88000 Mostar, Bosnia and Herzegovina; ivan.cavar@mef.sum.ba; 8 Department of Oncology and Hematology, General Hospital Pula, 52200 Pula, Croatia; 9 Faculty of Medicine, Juraj Dobrila University of Pula, 52100 Pula, Croatia; 10 Department of Gastroenterology, Clinical Hospital Center Rijeka, 51000 Rijeka, Croatia

**Keywords:** chemotherapy, chronotherapy, circadian rhythm, immunotherapy, oncology

## Abstract

Circadian rhythms regulate key biological processes central to cancer biology, including cell cycle control, DNA repair, metabolism, immune function, and drug pharmacokinetics. Experimental models consistently demonstrate marked time-of-day differences in the efficacy and toxicity of chemotherapy, targeted agents, and immunotherapies. Clinical studies of chronomodulated chemotherapy—particularly with fluoropyrimidines, platinum compounds, and anthracyclines—show reproducible reductions in treatment-related toxicity, while effects on survival outcomes remain variable and often sex dependent. Emerging clinical evidence also suggests that timing of immune checkpoint inhibitor administration may influence progression-free and overall survival across several tumor types. However, translation into routine oncology practice has been limited by interindividual variability in circadian phase, tumor-specific disruption of clock function, lack of validated biomarkers, and logistical constraints of time-specific drug delivery. Advances in circadian phenotyping, wearable monitoring, and adaptive dosing technologies now offer feasible pathways toward individualized, biology-driven treatment timing. This narrative review critically evaluates mechanistic, preclinical, and clinical evidence for chronotherapy across oncological treatment modalities and examines challenges and opportunities for its integration into precision cancer care.

## 1. Introduction

Cancer remains a leading cause of morbidity and mortality worldwide [1]. Over the past decade, major therapeutic advances—including targeted drugs, immunotherapies, gene and cell-based therapies, and innovative drug delivery systems—have markedly improved outcomes across many tumor types. Yet treatment resistance, tumor heterogeneity, and therapy-related toxicity continue to limit long-term success. These challenges highlight the need for strategies that not only target the right pathway but also deliver therapy at the right time [2].

The circadian clock is a fundamental biological system that generates near-24 h rhythms in gene expression, metabolism, and cellular behavior across almost all tissues [3,4,5]. This timing system is driven by molecular clock genes—such as BMAL1, PER, and CRY—and synchronized by environmental cues including light, feeding, and hormones. Circadian rhythms coordinate many physiological processes relevant to cancer biology, including cell division, DNA repair, immune surveillance, and metabolism. When these rhythms are disrupted—by genetic, behavioral, or environmental factors—physiological integrity declines, creating conditions that favor tumor initiation, progression, and therapeutic resistance [2,6].

At the molecular level, circadian regulators interact with key oncogenic pathways, such as p53, MYC, and PI3K/AKT, shaping tumor growth and response to stress. Experimental models have shown striking time-of-day differences in the efficacy and toxicity of chemotherapy, radiotherapy, and targeted agents [2,6]. In clinical settings, several studies in solid tumors have found that aligning treatment delivery with circadian phase can improve drug tolerability and, in some cases, enhance efficacy [7,8]. These observations reflect rhythmic variations in pharmacokinetics, DNA repair, hormone secretion, and tissue recovery. Together, they suggest that the timing of treatment represents a largely untapped dimension of therapeutic precision.

Despite experimental and translational evidence on chronotherapy benefits, the deliberate scheduling of therapy according to the body’s circadian rhythms in routine oncology remains limited. Barriers include wide interindividual variation in circadian timing, the absence of robust biomarkers, and the logistical demands of time-specific dosing. Recent advances, however, are transforming this landscape. Wearable sensors, blood-based transcriptomic clocks, organ-on-a-chip models, and artificial intelligence (AI)–guided dosing algorithms now allow real-time assessment of circadian phase and personalized, time-aware treatment planning [2,9].

**Figure 1:**
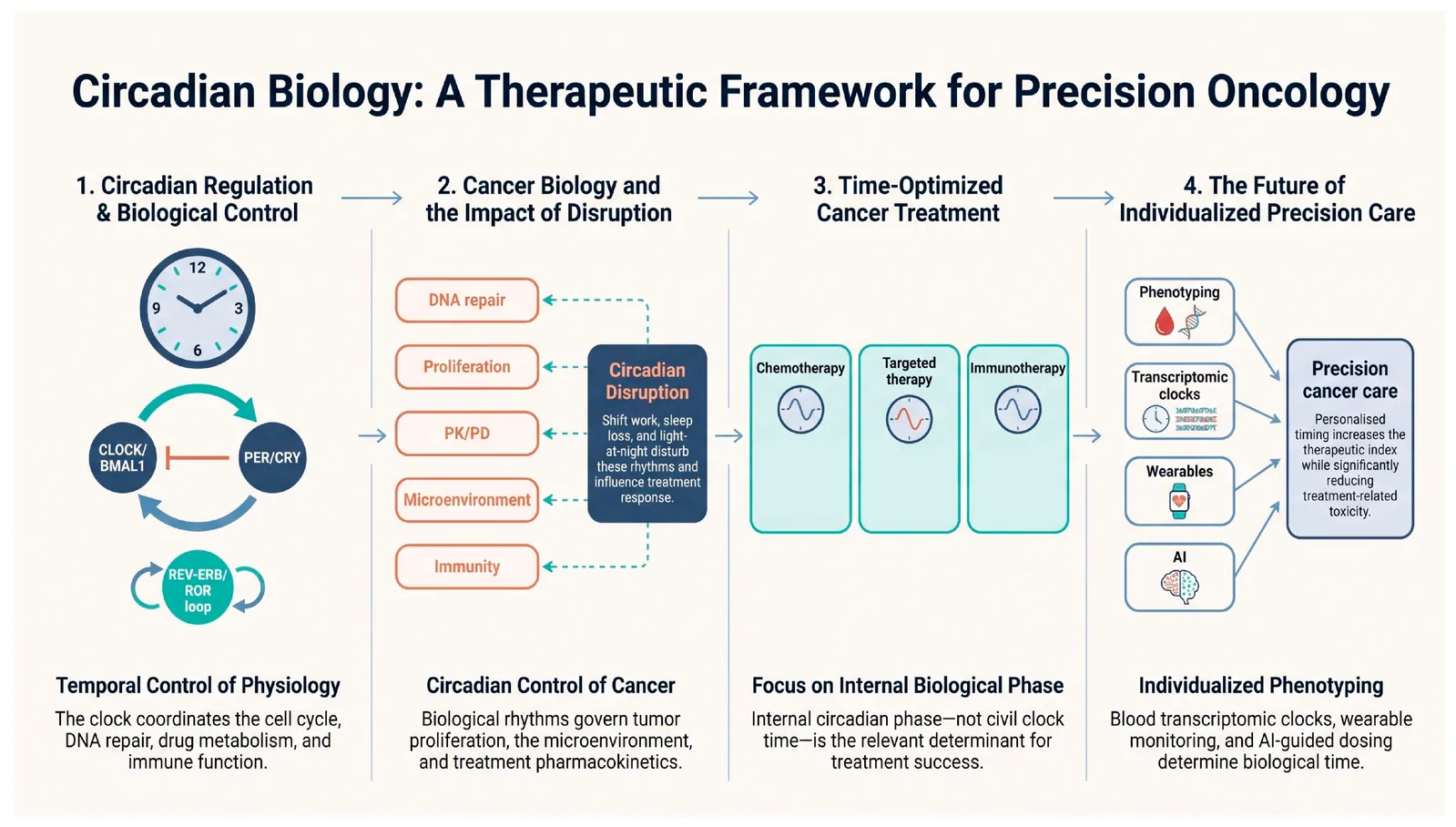
Circadian biology as a therapeutic framework for precision oncology. **Note:** The figure presents a conceptual overview of how circadian biology can be integrated into precision oncology. The endogenous molecular clock regulates fundamental processes relevant to cancer therapy, including cell-cycle progression, DNA repair, pharmacokinetics/pharmacodynamics, tumor biology, and antitumour immunity. Circadian disruption may impair these mechanisms and influence treatment response. Chronotherapy aims to align treatment administration with the patient’s internal biological phase to improve the therapeutic index by reducing toxicity while maintaining or enhancing efficacy. Emerging approaches, including circadian phenotyping, blood transcriptomic clocks, wearable technologies, and artificial intelligence-guided dosing, may facilitate individualized chronotherapy and support the implementation of precision cancer care. Current evidence is strongest for chemotherapy, whereas prospective validation remains necessary for targeted therapies and immunotherapy. **Abbreviations:** AI—artificial intelligence; BMAL1—Brain and Muscle ARNT-Like Protein 1; CLOCK—Circadian Locomotor Output Cycles Kaput; CRY—cryptochrome; DNA—deoxyribonucleic acid; PD—pharmacodynamics; PER—period; PK—pharmacokinetics; REV-ERB—reverse erythroblastosis virus α/β nuclear receptors (NR1D1/NR1D2); ROR—retinoic acid receptor-related orphan receptor.

This review provides a comprehensive synthesis of current knowledge on how circadian regulation shapes cancer biology and therapeutic response (Figure 1). We first outline the molecular and systemic architecture of the circadian clock and its intersection with oncogenic pathways. We then critically appraise mechanistic, preclinical, and clinical evidence supporting the role of treatment timing in modulating efficacy and toxicity across cancer types. Finally, we examine emerging technologies that could enable integration of biological time into precision oncology. By reframing therapeutic optimization through the lens of circadian biology, chronotherapy offers a promising route to enhance efficacy, reduce toxicity, and personalize cancer care.

## 2. Methods

This article was designed as a narrative review to integrate mechanistic, translational, and clinical evidence regarding the role of circadian biology in cancer development, therapeutic response, and the application of chronotherapy across chemotherapy, targeted therapy, and immunotherapy. Given the multidisciplinary and hypothesis-driven nature of this rapidly evolving field, encompassing oncology, circadian biology, pathophysiology, molecular medicine, pharmacology, and precision medicine, the aim was to provide a comprehensive conceptual synthesis rather than perform quantitative evidence pooling or formal systematic evaluation.

A targeted literature search was conducted using PubMed and Scopus databases, with additional relevant studies identified through manual screening of reference lists from key reviews, systematic reviews, meta-analyses, and clinical investigations. The search included publications available up to 1 January 2026 and combined controlled vocabulary and free-text terms related to circadian rhythms, chronotherapy, cancer, chemotherapy, targeted therapy, immunotherapy, molecular clock genes, melatonin, drug metabolism, and precision oncology. No predefined exclusion criteria based solely on publication type were applied due to the hypothesis-driven nature of the topic; therefore, experimental studies, preclinical models, observational studies, clinical trials, systematic reviews, meta-analyses, and relevant expert publications were considered. Publications were excluded if they were not available in English, lacked adequate methodological reporting according to evidence-based medicine principles, were of insufficient quality, or were outside the scope of circadian regulation and cancer therapy. All identified publications underwent initial title and abstract screening, followed by full-text assessment when considered relevant. Evidence interpretation followed evidence-based medicine principles, considering the hierarchy of evidence and giving greater emphasis to high-quality clinical studies while using mechanistic and preclinical findings primarily to support biological plausibility. Reference selection and interpretation were performed through multidisciplinary author consensus involving expertise in oncology, internal medicine, clinical pharmacology, clinical pharmacy, pathophysiology, immunology, and related biomedical fields; disagreements regarding relevance or interpretation were resolved through discussion within the team.

As a narrative review, this work did not follow formal PRISMA methodology, and no quantitative meta-analysis or standardized risk-of-bias assessment was performed. Nevertheless, predefined search procedures, transparent selection principles, and evidence-based interpretation were applied to provide a structured and clinically relevant overview of current knowledge and future directions in oncology chronotherapy.

## 3. The Biological Basis of Chronotherapy in Oncology

Several chronobiological terms used throughout this review require distinction. Chronopharmacology examines how biological timing modifies drug pharmacokinetics, pharmacodynamics, efficacy, and toxicity, whereas chronotherapy applies this knowledge clinically to optimize treatment timing. Chronomodulated infusion refers to programmable drug delivery in which the infusion rate varies over a predefined 24 h schedule rather than remaining constant. Circadian phase denotes an individual’s position within the endogenous approximately 24 h cycle and should not be equated with local civil clock time. In experimental studies conducted under a light–dark schedule, zeitgeber time (ZT) expresses time relative to an entraining cue, conventionally with ZT0 indicating lights-on and ZT12 lights-off. Circadian time (CT), by contrast, describes subjective biological time under constant or free-running conditions. The term diurnal variation refers to an observed change across the day under ordinary environmental and behavioral conditions; such variation may reflect endogenous circadian regulation, sleep–wake behavior, feeding, activity, light exposure, or a combination of these factors. Accordingly, external clock time is used in this review only as a study-specific operational marker and not as a universal equivalent of internal biological time [10,11,12].

At the cellular level, time is generated by self-sustaining molecular circuits that govern gene expression, division, and quiescence. These oscillatory systems are fundamental organizers of physiology, enabling anticipation and adaptation to environmental change [3,13]. Beyond the core circadian clock, additional oscillators regulate mitochondrial and cytoskeletal dynamics, interfacing with cell cycle rhythms [14]. Disruption of this architecture, by genetic, metabolic, or environmental stress, decouples cellular processes and destabilizes tissue homeostasis [13]. The molecular clock thus operates through interlocked transcriptional loops that sustain coherence under fluctuating conditions [15,16]. In cancer, this temporal order erodes early, as malignant cells progressively detach from systemic rhythmic control.

Circadian oscillations in mammals arise from interlocked transcriptional–translational feedback loops (TTFLs), where core transcription factors activate their own inhibitors to generate rhythmic gene expression and chromatin remodeling. The positive limb, formed by CLOCK (or NPAS2) and BMAL1, drives transcription of Period (Per1–3) and Cryptochrome (Cry1–2) genes, whose protein products dimerize and inhibit CLOCK–BMAL1 activity, closing the loop [17,18,19]. An additional regulatory loop is formed by REV-ERBα/β repressors and ROR activators, which act at ROR response elements within the Bmal1 promoter and contribute to the rhythmic regulation of Bmal1 transcription [20,21], while post-translational phosphorylation and ubiquitin-mediated degradation of clock proteins fine-tune period length and amplitude [17,18].

At the chromatin level, CLOCK–BMAL1 activation induces rhythmic recruitment of RNA polymerase II and histone acetylation (H3K9ac, H3K27ac), followed by deacetylation and methylation (H3K9me) during the repressive phase, producing alternating permissive and silent chromatin states [22,23,24]. Mathematical and genome-wide studies suggest that the interconnected positive and negative feedback loops of the mammalian circadian clock can be described using a *repressilator-like dynamical motif* as a simplified mathematical framework, rather than as the molecular architecture of the circadian clock itself. This conceptual model helps explain the generation of self-sustained oscillations and coordinated rhythmic transcription across large genomic domains [25].

Epigenetic oscillations link cellular metabolism to chromatin dynamics through the interlocked CLOCK/BMAL1–NAMPT–NAD^+^–SIRT1 feedback loop [26,27,28]. CLOCK/BMAL1 binds E-box elements within the Nampt promoter and rhythmically induces NAMPT, the rate-limiting enzyme of the NAD^+^ salvage pathway, thereby generating corresponding oscillations in intracellular NAD^+^ availability. Because SIRT1 is an NAD^+^-dependent deacetylase, these metabolic oscillations produce rhythmic changes in SIRT1 activity. SIRT1 is recruited to CLOCK/BMAL1-regulated promoters and deacetylates histone H3 and BMAL1, modulating chromatin accessibility and CLOCK/BMAL1-dependent transcription. SIRT1 also deacetylates PER2, promoting its turnover and thereby feeding cellular metabolic status back into the negative limb of the molecular clock. This interlocked transcriptional–enzymatic circuit couples energy availability to circadian gene expression and epigenetic regulation [26,27,28,29]. Additional chromatin regulators, including MLL1 and HDAC3, further shape rhythmic histone modification and time-dependent expression of metabolic and proliferative genes [30,31]. In cancer, disruption of clock control may uncouple NAD^+^ metabolism from normal temporal regulation, whereas sustained NAMPT-dependent NAD^+^ regeneration can support SIRT1- and PARP-dependent survival, cancer stem-cell properties, and treatment resistance [32]. The circadian clock coordinates cell cycle progression with DNA repair and metabolic state. Key regulators such as CDKs, WEE1, and p53 oscillate rhythmically, ensuring mitosis occurs only under genomic stability. WEE1 peaks at the circadian nadir of mitotic activity to restrain CDK1 and prevent premature G2/M transition [33]. PER2 rhythmically interacts with p53 and MDM2, stabilizing p53 and synchronizing growth arrest with repair. When circadian control is disrupted by shift work or chronic misalignment, these oscillations collapse, checkpoints fail, and cells divide with unrepaired DNA, accelerating mutation accumulation and tumorigenesis [33].

Alignment of circadian rhythms with DNA repair pathways optimizes genome maintenance and cellular defense against genotoxic stress. Core repair systems (nucleotide excision, base excision, and homologous recombination) exhibit time-of-day variation, driven by clock-controlled enzymes such as XPA and OGG1 [34]. XPA peaks during the active phase in mice, maximizing removal of cisplatin-induced DNA adducts through transcriptional and post-translational regulation [35]. This rhythmicity aligns repair with periods of highest DNA vulnerability, reducing replication errors and mutation rates. In tumors, mutations or epigenetic silencing of clock genes disrupt these oscillations, leading to desynchronized repair and genomic instability [2,36]. Disrupted temporal control alters sensitivity to DNA-damaging therapies, explaining time-dependent variation in cisplatin efficacy and toxicity [37]. Chronotherapy leverages these rhythms by matching treatment to the peak repair capacity of normal tissues, thereby improving therapeutic selectivity and minimizing damage [2].

Disruption of circadian-regulated metabolism (including rhythmic translation, proteasomal turnover, autophagy, ribosomal biogenesis, and mTOR signaling) uncouples biosynthetic and catabolic pathways from temporal control, undermining cellular homeostasis. In normal tissues, the clock aligns protein synthesis, energy flux, and detoxification with repair and stress defense, preserving proteostasis and redox balance [38].

In cancer, oncogenic MYC activation abolishes rhythmic expression in most oscillating genes, enforcing continuous ribosomal and mitochondrial biogenesis and a persistent anabolic drive [39]. This metabolic rigidity, reinforced by hyperactive mTOR signaling, suppresses autophagy and blunts adaptive responses to stress [38]. While such reprogramming supports uncontrolled growth, it also creates therapeutic vulnerabilities: tumors locked in chronic anabolism are more sensitive to agents that disrupt energy production or biosynthetic flux [40].

Normal tissues, by contrast, retain circadian detoxification and repair rhythms, showing time-of-day differences in drug handling and tolerance. The absence of such oscillations in tumors limits the efficacy of chronomodulated therapy, emphasizing the need for approaches that exploit non-circadian metabolic weaknesses or integrate biomarkers of clock and metabolic status into treatment design [2].

The tumor microenvironment contains autonomous circadian clocks within stromal, endothelial, and immune cells that rhythmically regulate angiogenesis, vascular permeability, and immune surveillance. Endothelial clocks control daily variation in ICAM-1 and VCAM-1 expression, influencing lymphocyte trafficking and drug delivery [41]. CD8^+^ T cells also follow circadian cues, with oscillations in infiltration and effector activity that shape antitumor immunity and response to checkpoint blockade. Chronic inflammation disrupts these local rhythms, promoting aberrant angiogenesis and weakening immune coordination. Aligning therapy with periods of maximal vascular access and immune function may enhance chronotherapeutic efficacy [41].

Mitochondria form a vital interface between circadian timing and cellular fate. Their fusion–fission dynamics follow ~24 h oscillations coordinated by clock factors such as BMAL1 and PERIOD, aligning mitochondrial activity with sleep–wake and feeding cycles to optimize energy production and oxidative defense [42]. Circadian control extends to genes encoding respiratory chain components, antioxidant enzymes (SOD, GPx), and biogenesis regulators including PGC-1α and TFAM, ensuring rhythmic mitochondrial turnover and redox balance [43].

In cancer, these oscillations are blunted: mitochondria remain fragmented, mitophagy is impaired, and respiratory activity becomes constitutive, sustaining chronic ROS signaling and oncogenic activation [44]. Loss of mitochondrial rhythmicity deprives tumor cells of restorative “rest” phases, fostering oxidative stress and therapy resistance. Restoring these rhythms, pharmacologically or through timed behavioral interventions, may enhance the selectivity of redox-targeted therapies and improve the resilience of healthy tissues to treatment-induced injury [45].

Hormonal and neuroendocrine rhythms, particularly those of cortisol, melatonin, insulin, and thyroid hormones, synchronize peripheral clocks with the central pacemaker, coordinating metabolism, proliferation, and immune activity [46,47]. Melatonin, secreted predominantly during the biological night, exerts oncostatic effects through multiple receptor-dependent and receptor-independent mechanisms. MT1 and MT2 are predominantly Gi/o-coupled receptors, and their activation commonly reduces adenylyl cyclase activity and intracellular cAMP levels. However, downstream ERK/MAPK regulation is not uniformly inhibitory and depends on receptor subtype and expression, cellular context, melatonin concentration, exposure duration, and tumor type. In HepG2 hepatocarcinoma cells, melatonin reduced intracellular cAMP but increased ERK, p38, and JNK phosphorylation through a mechanism partially dependent on MT1, whereas in esophageal squamous cell carcinoma models it suppressed MEK/ERK and AKT signaling and enhanced sensitivity to 5-fluorouracil. Additional oncostatic actions include modulation of estrogen receptor signaling, cell-cycle control, apoptosis, metabolism, and angiogenesis [48,49,50]. Nocturnal light exposure disrupts melatonin synthesis, inducing circadian misalignment, insulin resistance, and inflammation that favor glycolytic metabolism and tumor growth [51]. Restoring dark-phase melatonin peaks through behavioral or pharmacologic means re-establishes circadian regulation of metabolic and immune pathways, enhancing therapeutic response and survival [52]. Thus, melatonin acts as an active modulator of tumor vulnerability and a promising target in cancer chronotherapy [46].

Circadian control of gene expression is mediated by rhythmic DNA looping, histone acetylation, and nuclear organization, which create temporal windows for transcriptional activity. Core clock proteins CLOCK and BMAL1 act as transcriptional activators and histone acetyltransferases, coordinating with deacetylases and remodelers to drive daily cycles of gene activation and repression [53]. Three-dimensional chromatin architecture further refines this rhythm through time-dependent enhancer-promoter interactions and cyclic RNA polymerase II recruitment [54]. In cancer, mutations and chromatin remodeling defects disrupt these oscillations, sustaining continuous expression of growth-promoting genes and abolishing rhythmic microRNA regulation of apoptosis and angiogenesis [53,54]. Chronotherapy seeks to restore this temporal order by resynchronizing epigenetic layers, re-establishing rhythmic transcription, and improving therapeutic efficacy [55].

The circadian organization of the immune system critically shapes antitumor defense and immunotherapy response. Cytotoxic lymphocytes, macrophages, and dendritic cells display rhythmic variation in cytokine release, antigen presentation, and effector function, regulated by intrinsic immune clocks and rhythmic endothelial cues within the tumor microenvironment [56]. Immune checkpoints, such as PD-1, CTLA-4, and PD-L1, oscillate across the circadian cycle, leading to time-dependent changes in tumor susceptibility [41]. Experimental studies and retrospective clinical observations suggest that immune-checkpoint inhibitor outcomes may vary according to administration time [41,57,58]. However, in the available clinical studies, terms such as “morning,” “early day,” and “afternoon” refer to investigator-defined local civil infusion time rather than to a directly measured circadian phase. These clock-time associations should therefore be interpreted as hypothesis-generating proxies for biological timing and not as evidence of a universally optimal morning treatment window [12]. Circadian disruption, marked by sleep loss, flattened cortisol rhythms, and melatonin deficiency, desynchronizes immunity, fosters inflammation, and reduces immunotherapy efficacy [59,60].

Circadian timing is a core organizer of cellular physiology, and its disruption reshapes cancer’s molecular circuitry. Misalignment between internal rhythms and environmental cues favors cells that evade temporal control, propagating desynchrony across tumor, immune, and metabolic systems. Understanding these dynamics enables therapies that exploit residual rhythmicity in healthy tissue while targeting cancer’s temporal vulnerabilities.

## 4. Chronotherapy in Chemotherapy: Mechanisms and Evidence

The application of chronotherapy in oncology aims to improve the therapeutic index of anticancer treatment by aligning drug administration with circadian variation in drug disposition, normal-tissue tolerance, and tumor susceptibility. Preclinical and clinical evidence indicates that administration time can modify the pharmacokinetics, toxicity, and, in selected settings, antitumor activity of cytotoxic agents. However, the magnitude and direction of these effects depend on the drug, treatment regimen, tumor type, sex, and the patient’s internal circadian phase [61,62,63]. Mechanistically, circadian clocks regulate drug-metabolizing enzymes and uptake and efflux transporters in the liver, intestine, and kidney, while also coordinating cell-cycle progression, DNA repair, apoptosis, and normal-tissue recovery [63,64]. The following subsections therefore evaluate chronotherapeutic evidence using drug- and regimen-specific preclinical and clinical studies.

The tumor suppressor p53 is functionally linked to BMAL1-dependent circadian regulation, and disruption of BMAL1 may impair p53-mediated stress responses [62]. BMAL1 has also been linked to regulation of the unfolded protein response (UPR), a cellular stress-adaptation pathway that can support tumor-cell survival under conditions such as nutrient deprivation and hypoxia by maintaining protein-folding capacity [65]. Circadian regulation of WEE1 links the molecular clock to G2/M checkpoint control, and WEE1 inhibitors are being investigated for their potential to enhance chemotherapy-induced DNA damage [66,67,68]. Beyond treatment timing, targeted drug-delivery strategies represent another approach to improving the therapeutic index by increasing drug exposure within tumors while limiting exposure of healthy tissues. Such spatial optimization of drug delivery is conceptually distinct from, but potentially complementary to, temporal optimization through chronotherapy [69]. At the disease level, circadian-clock dysregulation has been implicated in colorectal cancer progression and may alter the temporal organization of proliferative, metabolic, and treatment-response pathways [70].

DNA-repair capacity exhibits circadian variation, but its phase and amplitude depend on the repair pathway, tissue, species, and experimental conditions. In mice, nucleotide-excision repair of cisplatin-induced DNA adducts oscillates through circadian regulation of the rate-limiting repair factor XPA, with robust rhythms demonstrated in the brain and liver but not in the testis. Preclinical timing should therefore be interpreted relative to the reported light–dark schedule, zeitgeber time, or circadian time rather than directly translated into human civil clock hours. Accordingly, the therapeutic consequences of timing DNA-damaging treatment depend on the temporal relationship between drug exposure, repair capacity in the tumor and normal tissues, and the patient’s internal circadian phase, rather than on a universally applicable late-afternoon or nighttime window [12,34,35].

Melatonin, secreted predominantly during the biological night, represents another potential mediator of time-dependent treatment response. Experimental studies indicate that melatonin can modulate tumor-cell proliferation, apoptosis, autophagy, oxidative stress, and sensitivity to cytotoxic therapy, although these effects vary according to cellular and tumor context [71,72,73]. Adjunctive melatonin has also been investigated during chemotherapy and radiotherapy, but the available clinical evidence should be regarded as preliminary rather than sufficient to support routine oncological use [74]. Exposure to light during the biological night can suppress the nocturnal melatonin signal. In a mechanistic xenograft study, melatonin-depleted blood collected from women following nighttime light exposure stimulated tumor metabolism and growth compared with melatonin-rich nocturnal blood [75]. Epidemiological findings are less consistent. A prospective analysis from the Nurses’ Health Study II identified a modest association between higher residential outdoor light-at-night exposure and invasive breast cancer risk, whereas a large prospective UK cohort found no association between self-reported domestic light-at-night exposure and subsequent breast cancer. Light at night should therefore be considered a potential circadian-disrupting exposure rather than an established independent cause of cancer [76,77].

### 4.1. Evidence in Treatment with 5-Fluorouracil

An important example of the role of the circadian timing system can be seen in studies of the pharmacokinetics and pharmacodynamics of dihydropyrimidine dehydrogenase (DPD), the enzyme that metabolizes 5-FU and is hence widely implicated in cancer treatment. DPD activity and 5-FU pharmacokinetics exhibit diurnal variation, although the phase and amplitude of these patterns show substantial interindividual variability. Recent physiologically based pharmacokinetic modeling has therefore emphasized the potential need to personalize chronomodulated 5-FU infusion according to the individual DPD chronotype [61,70,78].

Although much of the mechanistic evidence derives from nocturnal rodent models, preclinical studies indicate that 5-fluorouracil (5-FU) toxicity and antitumor activity can vary according to the phase of the experimentally defined light–dark cycle. Such findings should not be directly translated into equivalent human civil clock hours because laboratory time does not necessarily represent the same biological phase across species. Early clinical studies subsequently evaluated predefined chronomodulated 5-FU schedules. In a phase I study, a 14-day circadian infusion of 5-FU and leucovorin initially peaking at approximately 03:00–04:00 enabled greater dose intensity than flat-rate infusion; among patients who developed clinically relevant toxicity, shifting the peak to approximately 21:00–22:00 further improved tolerability and informed the recommended schedule for subsequent studies. A phase I–II study in advanced pancreatic cancer similarly demonstrated the feasibility of dose-escalated 5-FU infusion peaking at 04:00. In metastatic colorectal cancer, randomized comparison of chronomodulated and constant-rate oxaliplatin, 5-FU, and folinic acid delivery showed that chronomodulation, with 5-FU and folinic acid delivery peaking at 04:00, permitted higher dose intensity and substantially reduced severe mucosal toxicity. In a separate pancreatic-cancer chemoradiation pilot study, an eight-hour escalating–de-escalating 5-FU infusion peaking at 22:00 was feasible and was not associated with grade 3–4 hematological toxicity. However, these schedules were defined according to population-level local clock time and did not assess each patient’s endogenous circadian phase [61,79,80,81,82]. Results from larger human cohorts have been mixed: most studies report reduced toxicity with chronomodulated 5-FU but no improvement in oncological outcomes [83]. Conversely, some investigators have found no difference in toxicity between constant-rate and circadian infusions, possibly due to substantial inter- and intra-patient variability in 5-FU plasma concentrations, particularly in men [84,85]. Differences in gender may also play a role in chronomodulation [86].

### 4.2. Evidence in Treatment with Platinum Compounds

The chronomodulated delivery and metabolism of platinum-based chemotherapies has been explored as a strategy to enhance efficacy and reduce adverse effects. Several studies suggest that administering these drugs in the evening can reduce nephrotoxicity and hematological side effects. A prospective randomized trial in head and neck cancer reported better disease control and a more favorable toxicity profile when cisplatin was administered at 18:00 h [87]. Similar findings were observed in the trial by Zhang et al., which evaluated chronomodulated cisplatin infusion (peaking at 16:00 h) versus flat intermittent infusion combined with intensity-modulated radiation therapy in patients with locoregionally advanced nasopharyngeal carcinoma; the chronomodulated group experienced fewer adverse effects, although overall survival (OS) and progression-free survival (PFS) were unchanged [88]. Gou et al. reported comparable results, further supporting the toxicity-sparing effect of chronochemotherapy in nasopharyngeal carcinoma [89].

In non-small cell lung cancer, chronotherapy has similarly been associated with significantly lower rates of hematological toxicities, such as grade 3–4 leukopenia and neutropenia, as well as reduced gastrointestinal adverse effects, when cisplatin was administered at 18:00 h [90].

In a randomized multicenter trial involving 186 patients with previously untreated metastatic colorectal cancer, chronomodulated administration of oxaliplatin, 5-fluorouracil, and folinic acid produced a higher objective response rate than constant-rate infusion (51% vs. 29%, *p* = 0.003) and prolonged median time to treatment failure (6.4 vs. 4.9 months, *p* = 0.006). Chronomodulated treatment also markedly reduced severe mucosal toxicity (14% vs. 76%, *p* < 0.0001) and functional impairment due to peripheral sensory neuropathy (16% vs. 31%, *p* < 0.01). However, median OS and 3-year survival did not differ significantly between the groups, indicating that the principal demonstrated benefits were improved tumor response and tolerability rather than prolonged survival [91]. These findings are consistent with most studies, which show clearer benefits for toxicity than for survival. In a multicenter phase III trial involving 564 patients with previously untreated metastatic colorectal cancer, four-day chronomodulated delivery of fluorouracil, leucovorin, and oxaliplatin (chronoFLO4) produced similar median OS to conventional two-day FOLFOX2 delivery in the overall population (19.6 vs. 18.7 months, *p* = 0.55). However, a significant sex–schedule interaction was observed (*p* < 0.001). Among men, chronoFLO4 was associated with a 25% reduction in the risk of death and longer median OS than FOLFOX2 (21.4 vs. 18.3 months, *p* = 0.02), whereas among women it was associated with a 38% increase in the risk of earlier death and shorter median OS (16.3 vs. 19.1 months, *p* = 0.03). These findings suggest that sex may modify the effect of chronomodulated treatment delivery, but this subgroup finding requires prospective validation [86].

### 4.3. Evidence in Treatment with Other Chemotherapeutic Agents

Doxorubicin has also been shown to exhibit circadian timing–dependent effects. Most of the available data come from trials in the 1990s, where Lévi et al. demonstrated that doxorubicin toxicity could be significantly reduced by administering doxorubicin in the early morning and cisplatin in the late afternoon [92]. Barrett et al. similarly reported that circadian-timed delivery of doxorubicin–cisplatin chemotherapy was reasonably well tolerated and produced notable response rates in patients with advanced or recurrent endometrial carcinoma [93]. However, a later randomized study found no improvement in oncological outcomes for circadian-timed cisplatin–doxorubicin in endometrial cancer [94]. Positive results were also reported in a small cohort of bladder cancer patients receiving chronomodulated doxorubicin [95]. Some of the benefits of doxorubicin chronomodulation may relate to a significant decrease in total body clearance when the drug is administered at 21:00 h, resulting in a longer elimination half-life and increased AUC, likely due to circadian changes in hepatic blood flow [96].

The commonly used gastrointestinal cancer drug irinotecan has also demonstrated time-of-day-dependent effects. Available data suggest that morning administration may be more beneficial for male patients, whereas afternoon dosing appears to reduce toxicity and improve effectiveness in female patients [62,97,98]. A systematic review and meta-analysis of seven randomized trials involving 1137 patients found no significant difference in objective response rate between chronomodulated and conventional chemotherapy. However, chronomodulated regimens were associated with lower hematological toxicity, whereas differences in gastrointestinal, neurological, and cutaneous toxicity were not statistically significant [83].

Temozolomide (TMZ), an oral alkylating agent widely used for glioblastoma, also appears to be influenced by circadian timing. In vitro studies in murine GBM models show that TMZ sensitivity varies according to treatment time, with the greatest growth inhibition occurring near the peak of BMAL1-luc expression [99,100]. Retrospective clinical data indicate that morning TMZ administration is associated with longer overall survival than evening dosing (1.43 years vs. 1.13 years), with the greatest benefit observed in patients with MGMT-methylated tumors [101]. However, a small randomized feasibility trial found no significant difference in overall survival between morning and evening temozolomide administration; nevertheless, the study was not powered to establish differences in survival outcome [102]. Therefore, the current evidence base remains small, and no definitive recommendation regarding TMZ timing can yet be made. Future research should also consider patients’ sleep–wake patterns, as some individuals assigned to evening dosing may have taken TMZ closer to their biological morning, potentially influencing outcomes [103].

To conclude, there is growing evidence of the importance of applying chemotherapy drugs in a chronomodulated way, primarily with results demonstrating reduced toxicity. These effects arise from circadian regulation of core clock genes (e.g., CLOCK, BMAL1) that control drug-metabolizing enzymes, transporters, and cellular stress responses. Because DNA repair and cell-cycle activity also vary by time of day, normal tissues may better withstand treatment during optimal circadian windows, while tumor cells—often rhythm-disrupted—remain sensitive. Melatonin further supports these rhythms with oncostatic and immune-modulating effects, and reduced nocturnal melatonin from light exposure has been linked to higher cancer risk. Together, these mechanisms provide the biological rationale for timed chemotherapy as a way to improve the therapeutic index in cancer care.

## 5. Chronotherapy in Targeted Therapy

Molecular targeted therapy selectively inhibits specific molecular pathways driving tumor growth, improving OS and PFS with fewer adverse effects than chemotherapy [104].

Circadian regulation of cell cycle proteins and apoptotic pathways can affect the sensitivity of cancer cells to targeted therapies. For example, PER1/2 induce G1 arrest/apoptosis by inhibiting PI3K/AKT/mTOR, while CRY1/2 degrade cMYC/E2F/TLK2, directly increasing sensitivity to CDK4/6 or MYC-targeted therapies during rhythmic peaks [8]. In principle, therapy may be most effective when administered at a time when expression or activity of the relevant target is greatest [4,105]. Integration of chronotherapy into targeted therapies could further optimize therapeutic outcomes [104].

Tyrosine kinase inhibitors (TKIs) work by blocking signaling pathways through receptor tyrosine kinases (RTKs) [106]. In a tumor-bearing mouse model maintained under a defined 12 h light–dark cycle, imatinib produced greater inhibition of tumor growth when administered during the early light phase than during the early dark phase. This dosing-time dependence paralleled stronger inhibition of tumor angiogenesis and PDGF-receptor activity. Because the study was conducted in nocturnal rodents, the light-phase finding should not be directly translated into a specific human clock-time recommendation [107]. Sunitinib also exhibits administration-time-dependent pharmacokinetic variation. In a prospective randomized crossover study, 12 patients received sunitinib at 08:00, 13:00, and 18:00 during successive treatment courses. Trough concentrations were significantly lower following morning administration than after afternoon or evening administration, whereas total systemic exposure, measured by the area under the concentration–time curve, did not differ significantly between dosing times. These findings suggest temporal variation in sunitinib elimination and potential relevance for therapeutic drug monitoring, but they do not establish that a particular dosing time improves antitumor efficacy, safety, or quality of life [108]. Although preclinical studies indicate time-of-day variation in sunitinib pharmacokinetics, clinical evidence supporting a specific administration window remains insufficient. The cited phase II studies evaluated continuous once-daily sunitinib treatment but did not prospectively compare morning and evening administration and therefore cannot establish a clinical chronotherapeutic effect. Dedicated randomized timing studies are required before a preferred administration phase can be proposed [109,110].

Hormone-modulating drugs typically work by blocking pro-tumor hormone signaling or by blocking the hormone’s function. Since endocrine therapies often cause significant systemic side effects that influence patient adherence, most trials in chrono-endocrine therapy are focused on reducing side effects. In the REaCT-CHRONO RCT study, the primary goal was to evaluate whether taking adjuvant endocrine therapy in the morning compared to the evening made a difference in side effects and quality of life. The study, however, showed no significant difference between the two groups (*p* = 0.086) [111].

Anti-angiogenic drugs inhibit the formation of new blood vessels within and around the tumor [112]. For instance, TNP-470 blocks tumor blood vessel growth by inhibiting the MetAP2 enzyme. Studies on mice show that its effectiveness and concentration in the body depend on the time of day, with optimal results during the early light phase in lab models. These findings suggest that timed dosing could improve treatment results and reduce side effects [113].

mTOR is a serine/threonine kinase that regulates cellular growth, metabolism, and survival and represents the principal pharmacological target of everolimus. Preclinical experiments indicate that the pharmacodynamic and toxic effects of everolimus vary according to circadian phase. Pilot observations in women with metastatic breast cancer have also suggested that morning administration may reduce selected adverse effects, particularly fatigue and metabolic disturbances. However, these clinical observations were reported from a small exploratory study and do not establish improved antitumor efficacy or a universally optimal administration time. The available evidence should therefore be regarded as hypothesis-generating and requires confirmation in adequately powered prospective timing trials [88,114,115]. Anti-EGFR drugs inhibit the tyrosine kinase activities of EGFR and HER2, blocking signals that promote tumor growth. Animal studies show that EGFR signaling peaks during the resting phase, while glucocorticoid levels rise in the active phase. Recent data in preclinical models with erlotinib show higher efficacy in inhibiting tumor growth when the drug is administered during the early light phase [116], aligning with the peak natural activity of EGFR and its downstream factors, AKT and MAPK [117].

While circadian rhythm molecules play a major role in optimizing treatment timing, they are also future targets for new targeted therapies in oncology. For example, pharmacological activation of REV-ERBα and REV-ERBβ receptors with synthetic agonists (SR9009 and SR9011) induces apoptosis in cells with selective cytotoxicity toward malignant cells and minimal toxicity to normal cells [118]. Complementing this direct targeting, pivotal research demonstrates that flavonoid-mediated activation of the BMAL1 pathway can reprogram the tumor’s internal clock to overcome resistance to tyrosine kinase inhibitors (TKIs), marking a new era where biological rhythms are manipulated to restore drug sensitivity [119].

## 6. Chronotherapy in Immunotherapy

Circadian clocks in immune cells (BMAL1, CLOCK, PER, CRY) regulate daily gene expression, lymphocyte migration via CXCR4/CCR7, and T-cell differentiation (Th17). The migration of dendritic cells into the draining lymph node and expression of molecules important for antigen presentation also occur in a circadian rhythm. The numbers of T and B cells in the blood vary over the day under the influence of cortisol and chemokines, and T-cell effector functions including priming, proliferation and IFNγ production differ in strength depending on the time of day [120].

Preclinical studies suggest that the effects of cytokine-based immunotherapy may vary according to administration time; for example, time-dependent responses to interferon-β have been reported in animal models [121]. Early clinical exploration of chrono-immunotherapy included chronomodulated interleukin-2 administration in patients with metastatic renal cell carcinoma. In a prospective phase I–II dose-escalation study involving 30 patients, three eight-hour infusion schedules were evaluated: 05:00–13:00, 13:00–21:00, and 21:00–05:00. However, the groups were markedly unequal, comprising 2, 15, and 13 patients, respectively. The study primarily established the feasibility and tolerability of chronomodulated interleukin-2 delivery and identified a maximum tolerated dose of 14.0 MIU/m^2^. Overall survival was not significantly associated with the administration schedule. Therefore, although the study supports the feasibility of interleukin-2 chronoinfusion, it does not establish an optimal treatment window [122]. Most clinical evidence linking immune-checkpoint inhibitor administration time to treatment outcomes is derived from retrospective cohort studies. In the retrospective, single-center longitudinal MEMOIR study of 299 patients with stage IV melanoma, each 20-percentage-point increase in the proportion of ICI infusions administered after 16:30 was associated with a higher hazard of death (HR 1.31, 95% CI 1.00–1.71; *p* = 0.046). In the propensity-score-matched analysis, receiving at least 20% of infusions after 16:30 was associated with shorter OS than receiving fewer evening infusions (median OS 4.8 years versus not reached; HR 2.04, 95% CI 1.04–4.00; *p* = 0.038) [58].

In a separate retrospective cohort of 121 patients with advanced melanoma, administration of all first four ICI infusions in the afternoon was associated with shorter OS than administration of at least one of the first four infusions in the morning (adjusted HR 2.40, *p* = 0.004). Because treatment timing was defined according to recorded local infusion time rather than directly measured circadian phase, this finding should be considered hypothesis-generating [57].

A multicenter retrospective analysis of 201 patients with metastatic renal cell carcinoma found that receiving at least 20% of ICI infusions before noon was associated with longer PFS (adjusted HR 0.70, 95% CI 0.50–0.98; *p* = 0.040) and OS (adjusted HR 0.57, 95% CI 0.33–0.98; *p* = 0.043). The analysis used local infusion time as the exposure variable, and initial treatment regimens included nivolumab, nivolumab plus ipilimumab, and pembrolizumab [123].

In a retrospective single-center cohort of 62 patients with recurrent or metastatic esophageal squamous cell carcinoma, receiving the first nivolumab infusion before 13:00 was associated with a higher response rate and longer PFS and OS. When administration timing during the first three months was considered, predominantly earlier administration was associated with a higher response rate and longer PFS, but not OS. No significant differences in response rate, PFS, or OS were observed when the timing of all treatment courses was analyzed [124].

Finally, a retrospective pilot cohort of 95 patients with metastatic non-small-cell lung cancer classified patients according to their median civil infusion time. Median PFS was longer among patients whose nivolumab infusions occurred predominantly between 09:27 and 12:54 than among those treated predominantly between 12:55 and 17:14 (11.3 vs. 3.1 months; *p* < 0.001), as was median OS (34.2 vs. 9.6 months; *p* < 0.001). Because the study was non-randomized and treatment groups were defined according to each patient’s median local infusion time, these large effect estimates require prospective confirmation [125].

Collectively, these observational studies demonstrate broadly consistent associations between earlier civil infusion times and favorable outcomes across several tumor types. However, their retrospective designs, heterogeneous treatment regimens and timing thresholds, potential residual confounding and scheduling-related biases, and absence of direct circadian-phase assessment preclude causal inference or the definition of a universally optimal morning treatment window [59,126].

Although preclinical studies consistently support the biological rationale for chrono-immunotherapy, current clinical evidence suggesting potential benefits of timed immune checkpoint inhibitor administration remains limited and inconsistent across cancer types and therapeutic settings. Reliable prospective randomized evidence supporting a clinical benefit of time-of-day immunotherapy or immunochemotherapy is still unavailable, as existing findings are derived predominantly from retrospective cohort analyses and small exploratory or pilot studies. These studies remain susceptible to residual confounding, treatment-scheduling bias, institutional workflow effects, heterogeneous treatment contexts, and imprecise characterization of patients’ internal circadian phase. Transparently registered and adequately powered pragmatic randomized trials are therefore required, with prospectively defined treatment-timing exposures, prespecified clinical endpoints, rigorous control of scheduling-related biases, and incorporation of objective or validated measures of internal circadian timing. Until such evidence becomes available, treatment-timing strategies should be regarded as hypothesis-generating rather than sufficiently established for routine oncology practice [12,126]. Representative clinical studies evaluating treatment timing across chemotherapy, endocrine therapy, and immune-checkpoint inhibition are summarized in Table 1. The reported administration windows refer to study-specific local clock time unless an endogenous circadian phase was directly assessed; therefore, they should not be interpreted as universally optimal treatment times.

## 7. Challenges, Future Directions, and Clinical Implications of Chronotherapy in Oncology

Current findings indicate that chronotherapy most reliably improves safety in chemotherapy, shows selective promise in targeted therapy, and may meaningfully influence efficacy in immunotherapy, underscoring the need for modality-specific implementation strategies.

Despite this rationale, translation into routine oncology practice remains constrained by biological heterogeneity, methodological limitations, and operational barriers. Interindividual variability in circadian phase—driven by genetics, sex, age, chronotype, comorbidities, and environmental disruption—complicates the use of fixed clock-time dosing, particularly in advanced cancer where circadian amplitude is often blunted. Thus, chronotype can be assessed using validated questionnaires such as the Morningness–Eveningness Questionnaire (MEQ) or Munich ChronoType Questionnaire (MCTQ), complemented by objective measures such as actigraphy-derived rest–activity patterns, which may help overcome limitations associated with questionnaire-based assessments when available [128]. Tumor-related factors further obscure predictability: oncogenic signaling, inflammation, hypoxia, and metabolic reprogramming frequently dismantle tumor-intrinsic clocks, limiting opportunities to exploit rhythmic vulnerabilities for efficacy while preserving benefits for normal tissue protection. Clinical trials have largely failed to account for this complexity, relying on externally defined dosing times without biological phase assessment and remaining underpowered to detect sex-specific or chronotype-specific effects. Multi-agent regimens introduce additional trade-offs, as optimal timing for efficacy and toxicity may diverge across drugs and organs. Practical constraints also persist, as infusion centers, pharmacy workflows, staffing models, and reimbursement structures are rarely designed to accommodate time-adapted delivery, dampening institutional willingness to adopt chronotherapy outside research settings.

Future progress depends on reframing chronotherapy as a personalized, data-driven component of precision oncology. Advances in circadian phenotyping, including transcriptomic and metabolomic clocks, alongside wearable-derived physiological rhythms, now permit estimation of internal biological time with clinically meaningful resolution. What is more, transcriptomic approaches such as the TimeSignature assay estimate circadian phase from rhythmic gene-expression patterns and represent a potential biomarker framework for future biologically guided treatment scheduling [129].

Internal circadian phase can be assessed at different levels of precision. Dim-light melatonin onset, determined from serial evening saliva or plasma samples collected under controlled dim-light conditions, remains the most established physiological phase marker. The timing of the core body-temperature minimum provides another endogenous marker but generally requires prolonged and carefully controlled monitoring. Sleep diaries, habitual sleep timing, validated morningness–eveningness or chronotype questionnaires, and actigraphy-derived rest–activity rhythms are more feasible in clinical practice but represent indirect estimates that may be influenced by social schedules, treatment-related fatigue, physical inactivity, and sleep disruption. Emerging blood-based transcriptomic and metabolomic algorithms may estimate internal phase from one or a small number of samples and could facilitate scalable circadian phenotyping, although their performance and generalizability require further validation in patients with cancer. Therefore, future oncology trials should report both local administration time and, whenever feasible, a measured or explicitly defined estimate of internal circadian phase [10,11,12,130,131,132]. When integrated with pharmacokinetic, pharmacodynamic, and toxicity data through artificial intelligence-based models, these tools enable adaptive scheduling that reflects each patient’s evolving circadian status rather than rigid external timepoints. Such approaches are particularly relevant for immunotherapy and complex combination regimens, where immune competence and organ vulnerability fluctuate dynamically. Digital health infrastructure, including programmable infusion systems, oral dosing platforms, and mobile applications, can operationalize time-adaptive therapy in both inpatient and outpatient settings. Moreover, combining chronotherapy with circadian-restorative interventions, such as melatonin supplementation, light management, and sleep optimization, may amplify benefits by stabilizing host rhythmicity.

To move the field forward, future trials must embed circadian biology into design and reporting. Routine documentation of administration time, circadian phase, sex, rest–activity patterns, and light exposure is essential, as is the development of adaptive, biomarker-guided trials that distinguish timing for efficacy from timing for toxicity. Standardized reporting frameworks and multicenter collaboration will be required to generate reproducible evidence capable of supporting guideline-level implementation. Collectively, these advances position chronotherapy not as a niche scheduling strategy, but as a scalable approach to improving the safety, selectivity, and—where biology permits—efficacy of modern cancer therapy.

## Figures and Tables

**Table 1:** Representative clinical studies of chronotherapy in oncology.

Study	Regimen and Cancer Type	Compared Timing or Schedule	Design and Sample Size	Effect on Toxicity or Tolerability	Effect on Response, PFS, or OS	Main Methodological Limitations
**Verma et al., 2014** [87]	Weekly cisplatin with radiotherapy; locally advanced head and neck squamous cell carcinoma	Cisplatin at 06:00 versus 18:00	Single-center randomized study; **n = 60**	Evening administration was associated with less upper gastrointestinal toxicity and numerically less severe hematological and mucocutaneous toxicity	Short-term tumor response numerically favored evening dosing; no mature PFS or OS analysis	Small sample; single center; only 6-month follow-up; no assessment of internal circadian phase
**Zhang et al., 2018** [88]	Cisplatin with intensity-modulated radiotherapy; locoregionally advanced nasopharyngeal carcinoma	Chronomodulated infusion peaking at 16:00 versus flat intermittent infusion	Randomized phase II trial; **n = 148**	Lower rates of nausea, vomiting, and oral mucositis with chronomodulated treatment; less disruption of measured immune parameters	No significant difference in 2-year OS, PFS, or distant-metastasis-free survival	Phase II study; relatively short median follow-up; clock time used without phase phenotyping
**Gou et al., 2018** [89]	Cisplatin, 5-FU, and folinic acid followed by radiotherapy; nasopharyngeal carcinoma	Cisplatin from 10:00 to 22:00 and 5-FU/folinic acid from 22:00 to 10:00 versus conventional infusion	Prospective randomized phase II study; **n = 60**	Lower leukopenia, thrombocytopenia, nausea, and vomiting with chronomodulation	Higher response after induction treatment; no significant difference in long-term OS	Small study; treatment conducted in 2003–2004; multiple drugs were simultaneously chronomodulated; limited external generalizability
**Li et al., 2015** [90]	Cisplatin-based chemotherapy; advanced NSCLC	Cisplatin at 18:00 versus routine/morning administration	Randomized controlled study; **n = 41**	Lower grade 3–4 leukopenia and neutropenia and less gastrointestinal toxicity with evening cisplatin	Similar response rate, 52.9% versus 50.0%; survival benefit not demonstrated	Very small and unequally sized groups; no adequately powered PFS or OS analysis
**Lévi et al., 1997** [91]	Oxaliplatin, 5-FU, and folinic acid; metastatic colorectal cancer	Five-day chronomodulated infusion versus constant-rate infusion	Randomized multicenter trial; **n = 186**	Markedly lower severe mucosal toxicity and less treatment-limiting peripheral neuropathy	Higher response rate, 51% versus 29%, and longer time to treatment failure; no OS difference	Some baseline imbalance; crossover to chronotherapy occurred in the control group; regimen-level comparison rather than isolated timing of one drug
**Giacchetti et al., 2006** [86]	ChronoFLO4 versus FOLFOX2; metastatic colorectal cancer	Four-day chronomodulated delivery versus conventional two-day delivery	Multicenter randomized phase III trial; **n = 564**	Different toxicity profiles, with diarrhea predominating during chronoFLO4 and neutropenia during FOLFOX2	No OS benefit in the total population; longer survival in men but shorter survival in women receiving chronoFLO4	Sex interaction requires cautious interpretation; the two regimens differed in duration and delivery pattern, not only clock time
**Gallion et al., 2003** [94]	Doxorubicin plus cisplatin; advanced or recurrent endometrial cancer	Doxorubicin at 06:00 and cisplatin at 18:00 versus standard-timed administration	Randomized phase III trial; **n = 342**	No significant overall improvement in toxicity; modest numerical reduction in leukopenia	No significant difference in response, PFS, or OS	Older chemotherapy regimen; treatment timing was not individualized; no biological-phase assessment
**Damato et al., 2021** [101]	Maintenance temozolomide; newly diagnosed glioblastoma	Morning versus evening administration	Retrospective single-center cohort; **n = 166**	Toxicity was not the principal comparative endpoint	Morning administration was associated with numerically longer OS, particularly in MGMT-methylated tumors	Non-randomized prescription timing; potential selection and treatment-adherence bias; subgroup findings; no direct phase measurement
**Atluri et al., 2020** [127]	Temozolomide; WHO grade III–IV high-grade glioma	Before 10:00 versus after 20:00	Prospective randomized feasibility study reported as a conference abstract; n = 28	No significant difference in quality of life; non-significant trend toward greater worsening of lymphocyte counts with morning dosing	No significant PFS or OS difference reported in patients with newly diagnosed glioblastoma	Small and heterogeneous cohort; preliminary conference abstract; insufficiently powered to assess survival efficacy
**Savard et al., 2025** [111]	Tamoxifen or aromatase inhibitor; hormone receptor-positive early breast cancer	Within 1 h of waking versus within 1 h of bedtime	Pragmatic multicenter open-label randomized trial; **n = 245**	No significant difference in endocrine-treatment tolerability, quality of life, adherence, or persistence	Survival outcomes were not evaluated	Open-label design; heterogeneous endocrine agents; designed for tolerability rather than recurrence or survival
**Qian et al., 2021** [58]	Ipilimumab, nivolumab, pembrolizumab, or combinations; stage IV melanoma	≥20% versus <20% of infusions after 16:30	Retrospective single-center longitudinal cohort with propensity-score matching; **n = 299**	No principal toxicity comparison	Later-infusion group had shorter OS, with HR for death 2.04; median OS 4.8 years versus not reached	Retrospective exposure definition; residual confounding; number and duration of received treatment cycles may influence timing classification; no phase measurement
**Yeung et al., 2023** [57]	Immune-checkpoint inhibitors; advanced melanoma	All first four infusions in the afternoon versus at least one first-four infusion in the morning	Retrospective cohort; **n = 121**	Not a principal endpoint	Exclusive afternoon dosing of the first four infusions was associated with shorter OS, adjusted HR 2.40	Small retrospective cohort; investigator-defined morning/afternoon categories; limited control of unmeasured confounding
**Patel et al., 2024** [123]	Nivolumab, pembrolizumab, or nivolumab/ipilimumab; metastatic renal cell carcinoma	≥20% versus <20% of infusions before noon	Multicenter retrospective cohort; **n = 201**	No principal toxicity comparison	Earlier-infusion group had longer PFS, adjusted HR 0.70, and OS, adjusted HR 0.57	Retrospective design; empirically selected timing threshold; heterogeneous ICI regimens; external clock time used as a proxy for phase
**Nomura et al., 2023** [124]	Nivolumab; recurrent or metastatic esophageal squamous cell carcinoma	First infusion before versus after 13:00; additional analysis of early infusions during the first 3 months	Retrospective single-center cohort; **n = 62**	No principal toxicity comparison	Earlier first infusion was associated with better response, PFS, and OS; predominantly earlier administration during the first 3 months was associated with better response and PFS, but not OS. No significant outcome differences were observed when all treatment courses were analyzed.	Small cohort; retrospective timing classification; multiple exploratory comparisons; potential treatment-duration bias
**Karaboué et al., 2022** [125]	Nivolumab; metastatic NSCLC	Predominantly 09:27–12:54 versus 12:55–17:14	Retrospective single-center pilot cohort; **n = 95**	Most toxicities were similar; some differences occurred in fatigue and skin toxicity	Median PFS 11.3 versus 3.1 months and median OS 34.2 versus 9.6 months in morning versus afternoon groups	Retrospective and non-randomized; large difference in number of treatment cycles between groups; substantial risk of time-dependent and residual confounding

Abbreviations: 5-FU, 5-fluorouracil; ICI, immune-checkpoint inhibitor; MGMT, O^6^-methylguanine-DNA methyltransferase; NSCLC, non-small-cell lung cancer; OS, overall survival; PFS, progression-free survival. Administration windows represent local civil time or study-defined treatment schedules and should not be interpreted as equivalent internal circadian phases.

## Data Availability

No new data were created or analyzed in this study. Data sharing is not applicable to this article.
